# 19-[(*E*)-4-Chloro­benzyl­idene]-16-(4-chloro­phen­yl)-2-hydr­oxy-1,11-diaza­hexa­cyclo­[15.3.1.0^2,10^.0^3,8^.0^10,17^.0^11,15^]henicosa-3(8),4,6-triene-9,18-dione

**DOI:** 10.1107/S1600536810018611

**Published:** 2010-05-26

**Authors:** Raju Suresh Kumar, Hasnah Osman, Aisyah Saad Abdul Rahim, Madhukar Hemamalini, Hoong-Kun Fun

**Affiliations:** a School of Chemical Sciences, Universiti Sains Malaysia, 11800 USM, Penang, Malaysia; b School of Pharmaceutical Sciences, Universiti Sains Malaysia, 11800 USM, Penang, Malaysia; c X-ray Crystallography Unit, School of Physics, Universiti Sains Malaysia, 11800 USM, Penang, Malaysia

## Abstract

In the title compound, C_32_H_26_Cl_2_N_2_O_3_, the piperidone ring adopts a chair conformation and the proline and pyrrolidine rings adopt envelope conformations. The indane ring system is essentially planar with an r.m.s. deviation of 0.011 Å for the non-H atoms. The dihedral angle between the two chloro-substituted benzene rings is 63.69 (10)°. Intra­molecular C—H⋯O and N—H⋯O hydrogen bonds may influence the mol­ecular conformation. In the crystal structure, mol­ecules are connected into layers by weak inter­molecular C—H⋯O hydrogen bonds.

## Related literature

For cyclo­addition reactions, see: Dondas *et al.* (2004 ▶); Boruah *et al.* (2007 ▶). For applications of pyrrolizines, see: Boruah *et al.* (2007 ▶); Dimmock *et al.* (2001 ▶); El-Subbagh *et al.* (2000 ▶); Lee *et al.* (2001 ▶); Liddell (1998 ▶). For puckering parameters, see: Cremer & Pople (1975 ▶). For the stability of the temperature controller used in the data collection, see: Cosier & Glazer (1986 ▶).
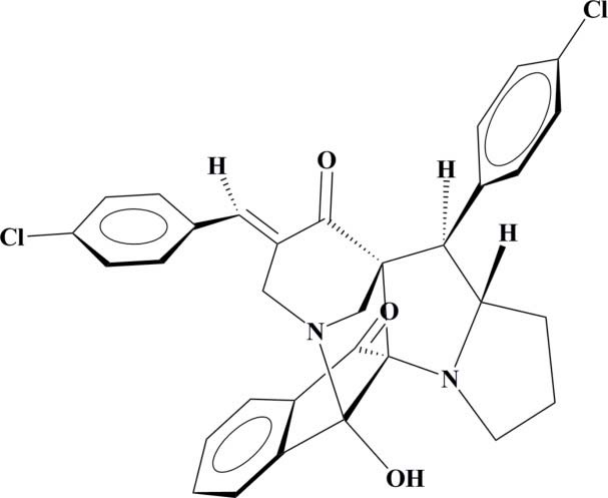


## Experimental

### 

#### Crystal data

C_32_H_26_Cl_2_N_2_O_3_
*M* *_r_* = 557.45
Monoclinic, 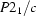

*a* = 14.603 (2) Å
*b* = 10.5701 (14) Å
*c* = 21.808 (2) Å
β = 130.094 (6)°
*V* = 2575.1 (5) Å^3^
*Z* = 4
Mo *K*α radiation
μ = 0.29 mm^−1^
*T* = 100 K
0.34 × 0.19 × 0.11 mm

#### Data collection

Bruker APEXII DUO CCD area-detector diffractometer
Absorption correction: multi-scan (*SADABS*; Bruker, 2009 ▶) *T*_min_ = 0.906, *T*_max_ = 0.969
27859 measured reflections
7631 independent reflections
5380 reflections with *I* > 2σ(*I*)
*R*_int_ = 0.045

#### Refinement

*R*[*F* ^2^ > 2σ(*F* ^2^)] = 0.057
*wR*(*F* ^2^) = 0.186
*S* = 1.06
7631 reflections
356 parameters
H atoms treated by a mixture of independent and constrained refinement
Δρ_max_ = 0.62 e Å^−3^
Δρ_min_ = −0.58 e Å^−3^

### 

Data collection: *APEX2* (Bruker, 2009 ▶); cell refinement: *SAINT* (Bruker, 2009 ▶); data reduction: *SAINT*; program(s) used to solve structure: *SHELXTL* (Sheldrick, 2008 ▶); program(s) used to refine structure: *SHELXTL*; molecular graphics: *SHELXTL*; software used to prepare material for publication: *SHELXTL* and *PLATON* (Spek, 2009 ▶).

## Supplementary Material

Crystal structure: contains datablocks global, I. DOI: 10.1107/S1600536810018611/lh5043sup1.cif

Structure factors: contains datablocks I. DOI: 10.1107/S1600536810018611/lh5043Isup2.hkl

Additional supplementary materials:  crystallographic information; 3D view; checkCIF report

## Figures and Tables

**Table 1:** Hydrogen-bond geometry (Å, °)

*D*—H⋯*A*	*D*—H	H⋯*A*	*D*⋯*A*	*D*—H⋯*A*
O2—H1*O*2⋯N2	0.86 (5)	1.97 (5)	2.623 (3)	133 (5)
C1—H1*A*⋯O3^i^	0.93	2.44	3.305 (3)	155
C22—H22*A*⋯O3	0.97	2.51	3.186 (3)	126
C23—H23*A*⋯O2^ii^	0.97	2.59	3.506 (3)	158

Symmetry codes: (i) 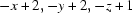; (ii) 
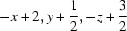
.

**Fig. 1.**
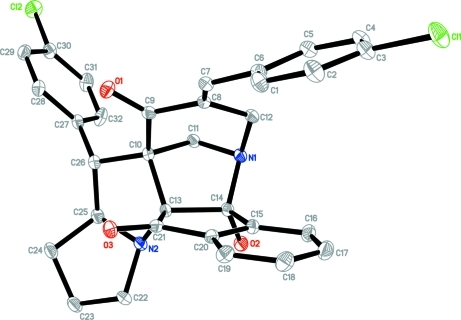
The asymmetric unit of the title compound, showing 30% probability displacement ellipsoids and the atom-numbering scheme (H atoms are omitted for clarity).

**Fig. 2.**
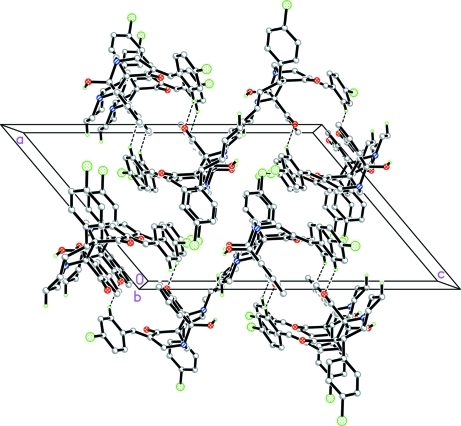
The crystal packing of the title compound, showing hydrogen-bonded (dashed lines) network. H atoms are not involing the hydrogen bond interactions are omitted for clarity.

## supplementary crystallographic information

### Comment

1,3-Dipolar cycloaddition of nonstabilized azomethine ylides with olefins represents one of the most convergent approaches for the construction of five membered heterocycles (Dondas *et al.*, 2004; Boruah *et al.* 2007). The pyrrolizine substructure occurs in many natural products of potential use in medicine and agriculture (Liddell, 1998). Heterocycles with piperidine sub-structures display important biological activities, such as cytotoxic (Dimmock *et al.*, 2001) and anticancer properties (El-Subbagh *et al.*, 2000) besides being useful as synthons in the construction of alkaloid natural products (Lee *et al.*, 2001). In view of the biological importance of aforementioned heterocycles, the crystal structure determination of the title compound was carried out and the results are presented herein.

The molecular structure of the title compound is shown in Fig.1. The piperidone (N1/C8–C12) ring adopts a chair conformation [Q = 0.608 (3) Å, Θ = 37.4 (3)°, φ= 59.8 (4)°; Cremer & Pople, 1975]. The proline ring (N2/C22–C25) and the five membered pyrrolidine ring (N1/C10/C11/C13/C14) adopt envelope conformations [puckering parameters Q = 0.398 (3) Å, φ = 269.4 (4)° and Q = 0.470 (3) Å, φ = 214.4 (3)° respectively]. The indane ring system is essentially planar with an rms deviation of 0.011 Å for the non-hydrogen atoms. The dihedral angle between the two chlorophenyl rings (C1–C6) and (C27–C32) is 63.69 (10)°. In the crystal structure, molecules are connected into layers by intermolecular weak C—H···O hydrogen bonds (Table 1, Fig. 2).

### Experimental

A mixture of 3,5-bis[(*E*)-(4-chlorophenyl)methylidene] tetrahydro-4-(1*H*)-pyridinone (0.100 g, 0.291 mmol), ninhydrin (0.052 g, 0.291 mmol) and proline (0.033 g, 0.291 mmol) were dissolved in methanol (10 mL) and refluxed for 30 minutes. After completion of the reaction as evident from TLC, the mixture was poured into water (50 mL). The precipitated solid was filtered and washed with water to afford the product which was recrystallised from ethyl acetate to reveal the title compound as yellow crystals.

### Refinement

Atom H1O2 was located in a difference Fourier map and refined freely. The remaining H atoms were positioned geometrically [O–H = 0.86 (4) Å and C–H = 0.93–0.98 Å] and were refined using a riding model, with *U*_iso_(H) = 1.2*U*_eq_(C).

### Crystal data

**Table:**

C_32_H_26_Cl_2_N_2_O_3_	*F*(000) = 1160
*M**_r_* = 557.45	*D*_x_ = 1.438 Mg m^−^^3^
Monoclinic, *P*2_1_/*c*	Mo *K*α radiation, λ = 0.71073 Å
Hall symbol: -P 2ybc	Cell parameters from 5137 reflections
*a* = 14.603 (2) Å	θ = 2.3–29.6°
*b* = 10.5701 (14) Å	µ = 0.29 mm^−^^1^
*c* = 21.808 (2) Å	*T* = 100 K
β = 130.094 (6)°	Block, yellow
*V* = 2575.1 (5) Å^3^	0.34 × 0.19 × 0.11 mm
*Z* = 4	

### Data collection

**Table:**

Bruker APEXII DUO CCD area-detector diffractometer	7631 independent reflections
Radiation source: fine-focus sealed tube	5380 reflections with *I* > 2σ(*I*)
graphite	*R*_int_ = 0.045
φ and ω scans	θ_max_ = 30.3°, θ_min_ = 1.8°
Absorption correction: multi-scan (*SADABS*; Bruker, 2009)	*h* = −19→20
*T*_min_ = 0.906, *T*_max_ = 0.969	*k* = −14→14
27859 measured reflections	*l* = −30→30

### Refinement

**Table:**

Refinement on *F*^2^	Primary atom site location: structure-invariant direct methods
Least-squares matrix: full	Secondary atom site location: difference Fourier map
*R*[*F*^2^ > 2σ(*F*^2^)] = 0.057	Hydrogen site location: inferred from neighbouring sites
*wR*(*F*^2^) = 0.186	H atoms treated by a mixture of independent and constrained refinement
*S* = 1.06	*w* = 1/[σ^2^(*F*_o_^2^) + (0.1017*P*)^2^ + 1.0123*P*] where *P* = (*F*_o_^2^ + 2*F*_c_^2^)/3
7631 reflections	(Δ/σ)_max_ < 0.001
356 parameters	Δρ_max_ = 0.62 e Å^−^^3^
0 restraints	Δρ_min_ = −0.58 e Å^−^^3^

### Special details

**Table:**

Geometry. All s.u.'s (except the s.u. in the dihedral angle between two l.s. planes) are estimated using the full covariance matrix. The cell s.u.'s are taken into account individually in the estimation of s.u.'s in distances, angles and torsion angles; correlations between s.u.'s in cell parameters are only used when they are defined by crystal symmetry. An approximate (isotropic) treatment of cell s.u.'s is used for estimating s.u.'s involving l.s. planes.
Refinement. Refinement of F^2^ against ALL reflections. The weighted R-factor wR and goodness of fit S are based on F^2^, conventional R-factors R are based on F, with F set to zero for negative F^2^. The threshold expression of F^2^ > 2σ(F^2^) is used only for calculating R-factors(gt) etc. and is not relevant to the choice of reflections for refinement. R-factors based on F^2^ are statistically about twice as large as those based on F, and R- factors based on ALL data will be even larger.

### Fractional atomic coordinates and isotropic or equivalent isotropic displacement parameters (Å^2^)

**Table:**

	*x*	*y*	*z*	*U*_iso_*/*U*_eq_	
Cl1	0.72105 (6)	0.37813 (6)	0.23348 (4)	0.03884 (18)	
Cl2	0.28180 (6)	1.55604 (6)	0.30038 (4)	0.03719 (17)	
O1	0.70242 (16)	1.14499 (14)	0.37668 (9)	0.0270 (3)	
O2	0.75301 (15)	0.85624 (15)	0.61320 (9)	0.0248 (3)	
O3	0.97349 (14)	1.06477 (14)	0.56290 (9)	0.0265 (3)	
N1	0.62861 (16)	0.87558 (16)	0.47338 (10)	0.0205 (3)	
N2	0.83265 (17)	1.08411 (16)	0.62301 (10)	0.0230 (4)	
C1	0.8080 (2)	0.7298 (2)	0.31990 (13)	0.0278 (5)	
H1A	0.8706	0.7859	0.3399	0.033*	
C2	0.8108 (2)	0.6108 (2)	0.29435 (14)	0.0315 (5)	
H2A	0.8744	0.5876	0.2966	0.038*	
C3	0.7182 (2)	0.5272 (2)	0.26543 (13)	0.0273 (5)	
C4	0.6213 (2)	0.5618 (2)	0.25928 (13)	0.0268 (5)	
H4A	0.5591	0.5050	0.2393	0.032*	
C5	0.6175 (2)	0.6819 (2)	0.28318 (12)	0.0245 (4)	
H5A	0.5511	0.7065	0.2774	0.029*	
C6	0.7127 (2)	0.76651 (19)	0.31604 (12)	0.0225 (4)	
C7	0.71498 (19)	0.89214 (19)	0.34519 (11)	0.0221 (4)	
H7A	0.7511	0.9561	0.3380	0.026*	
C8	0.67102 (18)	0.92557 (18)	0.38095 (11)	0.0201 (4)	
C9	0.69127 (19)	1.05940 (18)	0.40864 (11)	0.0210 (4)	
C10	0.69859 (19)	1.08168 (18)	0.48066 (11)	0.0193 (4)	
C11	0.59325 (19)	1.00825 (19)	0.46492 (12)	0.0214 (4)	
H11A	0.5842	1.0307	0.5039	0.026*	
H11B	0.5187	1.0251	0.4115	0.026*	
C12	0.61408 (19)	0.83699 (19)	0.40280 (11)	0.0214 (4)	
H12A	0.5292	0.8306	0.3573	0.026*	
H12B	0.6486	0.7534	0.4126	0.026*	
C13	0.80569 (19)	1.01008 (18)	0.55666 (11)	0.0198 (4)	
C14	0.75404 (19)	0.87571 (19)	0.55007 (11)	0.0207 (4)	
C15	0.8366 (2)	0.78312 (19)	0.55386 (12)	0.0222 (4)	
C16	0.8318 (2)	0.6517 (2)	0.55102 (13)	0.0269 (5)	
H16A	0.7728	0.6085	0.5472	0.032*	
C17	0.9170 (2)	0.5864 (2)	0.55402 (14)	0.0317 (5)	
H17A	0.9146	0.4985	0.5519	0.038*	
C18	1.0055 (2)	0.6494 (2)	0.56008 (15)	0.0321 (5)	
H18A	1.0621	0.6034	0.5625	0.039*	
C19	1.0108 (2)	0.7800 (2)	0.56257 (13)	0.0275 (5)	
H19A	1.0698	0.8228	0.5663	0.033*	
C20	0.92553 (19)	0.84543 (19)	0.55940 (11)	0.0216 (4)	
C21	0.91251 (19)	0.98351 (19)	0.56015 (11)	0.0209 (4)	
C22	0.9558 (2)	1.0914 (2)	0.70050 (12)	0.0278 (5)	
H22A	1.0140	1.0753	0.6936	0.033*	
H22B	0.9675	1.0305	0.7383	0.033*	
C23	0.9671 (2)	1.2266 (2)	0.72949 (13)	0.0307 (5)	
H23A	1.0495	1.2555	0.7637	0.037*	
H23B	0.9376	1.2333	0.7584	0.037*	
C24	0.8886 (2)	1.3011 (2)	0.65101 (13)	0.0286 (5)	
H24A	0.8644	1.3815	0.6580	0.034*	
H24B	0.9298	1.3161	0.6303	0.034*	
C25	0.7816 (2)	1.21395 (19)	0.59597 (12)	0.0237 (4)	
H25A	0.7231	1.2278	0.6036	0.028*	
C26	0.7192 (2)	1.22071 (18)	0.50652 (12)	0.0210 (4)	
H26A	0.7777	1.2545	0.5025	0.025*	
C27	0.6102 (2)	1.30567 (18)	0.45664 (12)	0.0221 (4)	
C28	0.5997 (2)	1.39566 (19)	0.40587 (13)	0.0251 (4)	
H28A	0.6608	1.4037	0.4036	0.030*	
C29	0.4999 (2)	1.4740 (2)	0.35843 (13)	0.0281 (5)	
H29A	0.4949	1.5344	0.3254	0.034*	
C30	0.4084 (2)	1.46082 (19)	0.36103 (14)	0.0277 (5)	
C31	0.4160 (2)	1.3725 (2)	0.41077 (16)	0.0322 (5)	
H31A	0.3542	1.3645	0.4124	0.039*	
C32	0.5155 (2)	1.2962 (2)	0.45792 (15)	0.0313 (5)	
H32A	0.5202	1.2371	0.4914	0.038*	
H1O2	0.782 (4)	0.925 (4)	0.640 (3)	0.077 (13)*	

### Atomic displacement parameters (Å^2^)

**Table:**

	*U*^11^	*U*^22^	*U*^33^	*U*^12^	*U*^13^	*U*^23^
Cl1	0.0295 (3)	0.0352 (3)	0.0469 (3)	−0.0032 (3)	0.0224 (3)	−0.0176 (2)
Cl2	0.0401 (4)	0.0299 (3)	0.0441 (3)	0.0115 (3)	0.0283 (3)	0.0032 (2)
O1	0.0343 (9)	0.0231 (7)	0.0303 (7)	0.0004 (7)	0.0238 (7)	0.0030 (6)
O2	0.0299 (8)	0.0265 (7)	0.0239 (7)	−0.0057 (7)	0.0199 (7)	−0.0006 (6)
O3	0.0230 (8)	0.0262 (7)	0.0299 (7)	−0.0067 (6)	0.0169 (7)	−0.0007 (6)
N1	0.0182 (8)	0.0210 (7)	0.0211 (7)	−0.0033 (7)	0.0121 (7)	−0.0020 (6)
N2	0.0238 (9)	0.0227 (8)	0.0191 (7)	−0.0050 (7)	0.0123 (7)	−0.0038 (6)
C1	0.0239 (11)	0.0296 (10)	0.0326 (10)	−0.0059 (9)	0.0195 (10)	−0.0061 (8)
C2	0.0252 (11)	0.0354 (12)	0.0370 (12)	−0.0043 (10)	0.0215 (11)	−0.0087 (9)
C3	0.0251 (11)	0.0282 (10)	0.0271 (10)	−0.0037 (9)	0.0161 (10)	−0.0078 (8)
C4	0.0225 (11)	0.0310 (10)	0.0256 (10)	−0.0063 (9)	0.0148 (9)	−0.0084 (8)
C5	0.0213 (10)	0.0292 (10)	0.0241 (9)	−0.0007 (9)	0.0151 (9)	−0.0035 (8)
C6	0.0213 (10)	0.0252 (9)	0.0198 (8)	−0.0007 (8)	0.0127 (8)	−0.0003 (7)
C7	0.0201 (10)	0.0222 (9)	0.0203 (8)	−0.0019 (8)	0.0114 (8)	−0.0008 (7)
C8	0.0171 (9)	0.0207 (8)	0.0173 (8)	−0.0009 (8)	0.0087 (8)	0.0004 (7)
C9	0.0185 (10)	0.0213 (9)	0.0191 (8)	0.0003 (8)	0.0103 (8)	−0.0001 (7)
C10	0.0198 (9)	0.0174 (8)	0.0206 (8)	−0.0017 (8)	0.0129 (8)	−0.0014 (6)
C11	0.0190 (10)	0.0217 (9)	0.0215 (9)	−0.0021 (8)	0.0121 (8)	−0.0017 (7)
C12	0.0189 (10)	0.0230 (9)	0.0197 (8)	−0.0031 (8)	0.0112 (8)	−0.0027 (7)
C13	0.0205 (10)	0.0180 (8)	0.0192 (8)	−0.0046 (8)	0.0120 (8)	−0.0017 (6)
C14	0.0205 (10)	0.0216 (9)	0.0206 (8)	−0.0049 (8)	0.0135 (8)	−0.0006 (7)
C15	0.0227 (10)	0.0212 (9)	0.0217 (9)	−0.0027 (8)	0.0138 (9)	0.0004 (7)
C16	0.0312 (12)	0.0218 (9)	0.0302 (10)	−0.0025 (9)	0.0209 (10)	0.0019 (8)
C17	0.0373 (13)	0.0221 (10)	0.0355 (11)	0.0032 (10)	0.0233 (11)	0.0025 (8)
C18	0.0318 (13)	0.0285 (10)	0.0381 (12)	0.0081 (10)	0.0235 (11)	0.0044 (9)
C19	0.0240 (11)	0.0299 (10)	0.0302 (10)	0.0031 (9)	0.0182 (10)	0.0047 (8)
C20	0.0195 (10)	0.0232 (9)	0.0196 (8)	−0.0006 (8)	0.0114 (8)	0.0020 (7)
C21	0.0175 (9)	0.0218 (9)	0.0181 (8)	−0.0003 (8)	0.0090 (8)	0.0021 (7)
C22	0.0248 (11)	0.0275 (10)	0.0207 (9)	−0.0058 (9)	0.0099 (9)	−0.0042 (8)
C23	0.0307 (12)	0.0320 (11)	0.0245 (10)	−0.0110 (10)	0.0155 (10)	−0.0087 (8)
C24	0.0311 (12)	0.0236 (9)	0.0283 (10)	−0.0085 (9)	0.0179 (10)	−0.0088 (8)
C25	0.0271 (11)	0.0212 (9)	0.0257 (9)	−0.0036 (8)	0.0184 (9)	−0.0041 (7)
C26	0.0229 (10)	0.0185 (8)	0.0241 (9)	−0.0028 (8)	0.0162 (9)	−0.0024 (7)
C27	0.0249 (10)	0.0178 (8)	0.0251 (9)	−0.0029 (8)	0.0168 (9)	−0.0032 (7)
C28	0.0296 (11)	0.0211 (9)	0.0316 (10)	−0.0019 (9)	0.0228 (10)	−0.0014 (7)
C29	0.0328 (12)	0.0220 (9)	0.0300 (10)	0.0007 (9)	0.0205 (10)	0.0023 (8)
C30	0.0308 (12)	0.0189 (9)	0.0344 (11)	0.0028 (9)	0.0214 (10)	−0.0028 (8)
C31	0.0331 (13)	0.0267 (10)	0.0492 (14)	0.0007 (10)	0.0322 (12)	0.0015 (9)
C32	0.0400 (14)	0.0227 (10)	0.0456 (13)	0.0027 (10)	0.0341 (12)	0.0068 (9)

### Geometric parameters (Å, °)

**Table:**

Cl1—C3	1.734 (2)	C13—C14	1.571 (3)
Cl2—C30	1.741 (2)	C14—C15	1.514 (3)
O1—C9	1.215 (2)	C15—C20	1.390 (3)
O2—C14	1.402 (2)	C15—C16	1.391 (3)
O2—H1O2	0.86 (4)	C16—C17	1.387 (4)
O3—C21	1.211 (3)	C16—H16A	0.9300
N1—C11	1.464 (3)	C17—C18	1.382 (4)
N1—C12	1.470 (3)	C17—H17A	0.9300
N1—C14	1.485 (3)	C18—C19	1.382 (3)
N2—C13	1.459 (2)	C18—H18A	0.9300
N2—C22	1.477 (3)	C19—C20	1.386 (3)
N2—C25	1.490 (3)	C19—H19A	0.9300
C1—C2	1.387 (3)	C20—C21	1.473 (3)
C1—C6	1.393 (3)	C22—C23	1.528 (3)
C1—H1A	0.9300	C22—H22A	0.9700
C2—C3	1.382 (3)	C22—H22B	0.9700
C2—H2A	0.9300	C23—C24	1.529 (3)
C3—C4	1.382 (3)	C23—H23A	0.9700
C4—C5	1.386 (3)	C23—H23B	0.9700
C4—H4A	0.9300	C24—C25	1.521 (3)
C5—C6	1.401 (3)	C24—H24A	0.9700
C5—H5A	0.9300	C24—H24B	0.9700
C6—C7	1.464 (3)	C25—C26	1.533 (3)
C7—C8	1.338 (3)	C25—H25A	0.9800
C7—H7A	0.9300	C26—C27	1.515 (3)
C8—C9	1.491 (3)	C26—H26A	0.9800
C8—C12	1.518 (3)	C27—C28	1.391 (3)
C9—C10	1.522 (3)	C27—C32	1.404 (3)
C10—C26	1.533 (3)	C28—C29	1.392 (3)
C10—C11	1.551 (3)	C28—H28A	0.9300
C10—C13	1.558 (3)	C29—C30	1.380 (4)
C11—H11A	0.9700	C29—H29A	0.9300
C11—H11B	0.9700	C30—C31	1.381 (3)
C12—H12A	0.9700	C31—C32	1.376 (3)
C12—H12B	0.9700	C31—H31A	0.9300
C13—C21	1.538 (3)	C32—H32A	0.9300
			
C14—O2—H1O2	103 (3)	C17—C16—C15	118.5 (2)
C11—N1—C12	109.42 (15)	C17—C16—H16A	120.8
C11—N1—C14	102.71 (15)	C15—C16—H16A	120.8
C12—N1—C14	114.90 (17)	C18—C17—C16	121.3 (2)
C13—N2—C22	120.99 (18)	C18—C17—H17A	119.3
C13—N2—C25	110.79 (15)	C16—C17—H17A	119.3
C22—N2—C25	109.58 (16)	C17—C18—C19	120.7 (2)
C2—C1—C6	121.0 (2)	C17—C18—H18A	119.7
C2—C1—H1A	119.5	C19—C18—H18A	119.7
C6—C1—H1A	119.5	C18—C19—C20	118.1 (2)
C3—C2—C1	119.3 (2)	C18—C19—H19A	121.0
C3—C2—H2A	120.4	C20—C19—H19A	121.0
C1—C2—H2A	120.4	C19—C20—C15	121.8 (2)
C2—C3—C4	121.0 (2)	C19—C20—C21	127.7 (2)
C2—C3—Cl1	119.65 (18)	C15—C20—C21	110.51 (19)
C4—C3—Cl1	119.31 (18)	O3—C21—C20	127.4 (2)
C3—C4—C5	119.4 (2)	O3—C21—C13	124.27 (19)
C3—C4—H4A	120.3	C20—C21—C13	108.33 (17)
C5—C4—H4A	120.3	N2—C22—C23	104.47 (19)
C4—C5—C6	120.7 (2)	N2—C22—H22A	110.9
C4—C5—H5A	119.7	C23—C22—H22A	110.9
C6—C5—H5A	119.7	N2—C22—H22B	110.9
C1—C6—C5	118.44 (19)	C23—C22—H22B	110.9
C1—C6—C7	119.0 (2)	H22A—C22—H22B	108.9
C5—C6—C7	122.6 (2)	C22—C23—C24	102.44 (16)
C8—C7—C6	127.4 (2)	C22—C23—H23A	111.3
C8—C7—H7A	116.3	C24—C23—H23A	111.3
C6—C7—H7A	116.3	C22—C23—H23B	111.3
C7—C8—C9	116.25 (19)	C24—C23—H23B	111.3
C7—C8—C12	125.83 (18)	H23A—C23—H23B	109.2
C9—C8—C12	117.63 (18)	C25—C24—C23	102.73 (17)
O1—C9—C8	122.60 (18)	C25—C24—H24A	111.2
O1—C9—C10	122.02 (18)	C23—C24—H24A	111.2
C8—C9—C10	115.37 (17)	C25—C24—H24B	111.2
C9—C10—C26	113.24 (16)	C23—C24—H24B	111.2
C9—C10—C11	107.07 (16)	H24A—C24—H24B	109.1
C26—C10—C11	119.53 (17)	N2—C25—C24	104.33 (18)
C9—C10—C13	112.20 (17)	N2—C25—C26	106.39 (15)
C26—C10—C13	104.45 (15)	C24—C25—C26	116.25 (18)
C11—C10—C13	99.52 (15)	N2—C25—H25A	109.9
N1—C11—C10	103.43 (16)	C24—C25—H25A	109.9
N1—C11—H11A	111.1	C26—C25—H25A	109.9
C10—C11—H11A	111.1	C27—C26—C10	115.95 (17)
N1—C11—H11B	111.1	C27—C26—C25	115.44 (17)
C10—C11—H11B	111.1	C10—C26—C25	103.79 (15)
H11A—C11—H11B	109.0	C27—C26—H26A	107.0
N1—C12—C8	114.75 (16)	C10—C26—H26A	107.0
N1—C12—H12A	108.6	C25—C26—H26A	107.0
C8—C12—H12A	108.6	C28—C27—C32	117.3 (2)
N1—C12—H12B	108.6	C28—C27—C26	120.2 (2)
C8—C12—H12B	108.6	C32—C27—C26	122.47 (18)
H12A—C12—H12B	107.6	C27—C28—C29	121.6 (2)
N2—C13—C21	115.59 (17)	C27—C28—H28A	119.2
N2—C13—C10	103.85 (16)	C29—C28—H28A	119.2
C21—C13—C10	115.64 (16)	C30—C29—C28	119.2 (2)
N2—C13—C14	112.50 (15)	C30—C29—H29A	120.4
C21—C13—C14	104.56 (16)	C28—C29—H29A	120.4
C10—C13—C14	104.36 (16)	C29—C30—C31	120.7 (2)
O2—C14—N1	108.68 (17)	C29—C30—Cl2	119.54 (17)
O2—C14—C15	111.33 (16)	C31—C30—Cl2	119.76 (19)
N1—C14—C15	114.98 (16)	C32—C31—C30	119.6 (2)
O2—C14—C13	110.56 (15)	C32—C31—H31A	120.2
N1—C14—C13	105.97 (15)	C30—C31—H31A	120.2
C15—C14—C13	105.12 (17)	C31—C32—C27	121.5 (2)
C20—C15—C16	119.7 (2)	C31—C32—H32A	119.2
C20—C15—C14	111.42 (17)	C27—C32—H32A	119.2
C16—C15—C14	128.9 (2)		
			
C6—C1—C2—C3	−0.7 (4)	C10—C13—C14—C15	124.24 (16)
C1—C2—C3—C4	2.1 (4)	O2—C14—C15—C20	−121.38 (18)
C1—C2—C3—Cl1	−179.90 (18)	N1—C14—C15—C20	114.48 (19)
C2—C3—C4—C5	−0.6 (3)	C13—C14—C15—C20	−1.6 (2)
Cl1—C3—C4—C5	−178.64 (17)	O2—C14—C15—C16	59.9 (3)
C3—C4—C5—C6	−2.3 (3)	N1—C14—C15—C16	−64.2 (3)
C2—C1—C6—C5	−2.1 (3)	C13—C14—C15—C16	179.6 (2)
C2—C1—C6—C7	178.8 (2)	C20—C15—C16—C17	0.1 (3)
C4—C5—C6—C1	3.6 (3)	C14—C15—C16—C17	178.8 (2)
C4—C5—C6—C7	−177.36 (19)	C15—C16—C17—C18	0.3 (3)
C1—C6—C7—C8	−146.5 (2)	C16—C17—C18—C19	−0.6 (4)
C5—C6—C7—C8	34.5 (3)	C17—C18—C19—C20	0.5 (3)
C6—C7—C8—C9	176.88 (19)	C18—C19—C20—C15	0.0 (3)
C6—C7—C8—C12	3.3 (3)	C18—C19—C20—C21	−179.2 (2)
C7—C8—C9—O1	27.9 (3)	C16—C15—C20—C19	−0.3 (3)
C12—C8—C9—O1	−158.0 (2)	C14—C15—C20—C19	−179.13 (18)
C7—C8—C9—C10	−151.40 (19)	C16—C15—C20—C21	179.02 (18)
C12—C8—C9—C10	22.7 (3)	C14—C15—C20—C21	0.2 (2)
O1—C9—C10—C26	0.8 (3)	C19—C20—C21—O3	0.8 (3)
C8—C9—C10—C26	−179.89 (18)	C15—C20—C21—O3	−178.47 (19)
O1—C9—C10—C11	134.7 (2)	C19—C20—C21—C13	−179.31 (19)
C8—C9—C10—C11	−46.0 (2)	C15—C20—C21—C13	1.4 (2)
O1—C9—C10—C13	−117.1 (2)	N2—C13—C21—O3	−58.2 (3)
C8—C9—C10—C13	62.2 (2)	C10—C13—C21—O3	63.4 (3)
C12—N1—C11—C10	−74.00 (18)	C14—C13—C21—O3	177.57 (18)
C14—N1—C11—C10	48.50 (18)	N2—C13—C21—C20	121.89 (17)
C9—C10—C11—N1	71.07 (18)	C10—C13—C21—C20	−116.49 (17)
C26—C10—C11—N1	−158.51 (16)	C14—C13—C21—C20	−2.35 (19)
C13—C10—C11—N1	−45.81 (17)	C13—N2—C22—C23	144.02 (19)
C11—N1—C12—C8	50.1 (2)	C25—N2—C22—C23	13.2 (2)
C14—N1—C12—C8	−64.8 (2)	N2—C22—C23—C24	−33.2 (2)
C7—C8—C12—N1	150.2 (2)	C22—C23—C24—C25	40.6 (2)
C9—C8—C12—N1	−23.3 (3)	C13—N2—C25—C24	−123.78 (18)
C22—N2—C13—C21	−21.8 (3)	C22—N2—C25—C24	12.2 (2)
C25—N2—C13—C21	108.51 (19)	C13—N2—C25—C26	−0.3 (2)
C22—N2—C13—C10	−149.50 (18)	C22—N2—C25—C26	135.68 (18)
C25—N2—C13—C10	−19.2 (2)	C23—C24—C25—N2	−32.7 (2)
C22—N2—C13—C14	98.2 (2)	C23—C24—C25—C26	−149.45 (19)
C25—N2—C13—C14	−131.49 (18)	C9—C10—C26—C27	78.5 (2)
C9—C10—C13—N2	154.42 (16)	C11—C10—C26—C27	−49.1 (2)
C26—C10—C13—N2	31.4 (2)	C13—C10—C26—C27	−159.14 (17)
C11—C10—C13—N2	−92.63 (17)	C9—C10—C26—C25	−153.81 (18)
C9—C10—C13—C21	26.7 (2)	C11—C10—C26—C25	78.6 (2)
C26—C10—C13—C21	−96.33 (19)	C13—C10—C26—C25	−31.4 (2)
C11—C10—C13—C21	139.66 (17)	N2—C25—C26—C27	148.12 (18)
C9—C10—C13—C14	−87.54 (19)	C24—C25—C26—C27	−96.2 (2)
C26—C10—C13—C14	149.42 (16)	N2—C25—C26—C10	20.1 (2)
C11—C10—C13—C14	25.41 (18)	C24—C25—C26—C10	135.8 (2)
C11—N1—C14—O2	88.01 (18)	C10—C26—C27—C28	−108.6 (2)
C12—N1—C14—O2	−153.27 (16)	C25—C26—C27—C28	129.7 (2)
C11—N1—C14—C15	−146.47 (17)	C10—C26—C27—C32	70.1 (3)
C12—N1—C14—C15	−27.7 (2)	C25—C26—C27—C32	−51.6 (3)
C11—N1—C14—C13	−30.83 (19)	C32—C27—C28—C29	0.4 (3)
C12—N1—C14—C13	87.89 (19)	C26—C27—C28—C29	179.10 (19)
N2—C13—C14—O2	−3.6 (2)	C27—C28—C29—C30	−0.9 (3)
C21—C13—C14—O2	122.61 (17)	C28—C29—C30—C31	0.9 (3)
C10—C13—C14—O2	−115.52 (18)	C28—C29—C30—Cl2	−178.67 (17)
N2—C13—C14—N1	114.01 (18)	C29—C30—C31—C32	−0.4 (4)
C21—C13—C14—N1	−119.79 (16)	Cl2—C30—C31—C32	179.21 (19)
C10—C13—C14—N1	2.1 (2)	C30—C31—C32—C27	−0.2 (4)
N2—C13—C14—C15	−123.83 (18)	C28—C27—C32—C31	0.2 (3)
C21—C13—C14—C15	2.37 (18)	C26—C27—C32—C31	−178.5 (2)

### Hydrogen-bond geometry (Å, °)

**Table:**

*D*—H···*A*	*D*—H	H···*A*	*D*···*A*	*D*—H···*A*
O2—H1O2···N2	0.86 (5)	1.97 (5)	2.623 (3)	133 (5)
C1—H1A···O3^i^	0.93	2.44	3.305 (3)	155
C22—H22A···O3	0.97	2.51	3.186 (3)	126
C23—H23A···O2^ii^	0.97	2.59	3.506 (3)	158

Symmetry codes: (i) −*x*+2, −*y*+2, −*z*+1; (ii) −*x*+2, *y*+1/2, −*z*+3/2.
